# Survival analysis of patients taking dabigatran who consulted an emergency department for acute bleeding: a first alarm

**DOI:** 10.1186/cc12285

**Published:** 2013-03-19

**Authors:** OA Arlet, EN Notebaert, JP Paquet, RD Daoust, MV Vincent, JC Chauny

**Affiliations:** 1 Hôpital du Sacré-Coeur de Montréal, Université de Montréal, Canada

## Introduction

The newly approved oral anticoagulant dabigatran has no effective antidote. We therefore suspected an overall increase in mortality in patients presenting to the emergency department (ED) with a bleeding complication on dabigatran compared with warfarin or aspirin.

## Methods

We conducted a *post hoc *analysis on a database of all patients admitted to a tertiary-care ED with any kind of bleeding or suspicion of one from March 2011 to August 2012 who were taking dabigatran, warfarin, or aspirin. The primary endpoint was long-term survival. Patients were censored at death or at the end of the study period (7 December 2012). We performed a Cox proportional hazard model, controlled for age, to calculate the hazard ratio (HR) for dabigatran versus warfarin and one for warfarin versus aspirin. Statistical significance was set at α = 0.05 and results are presented with 95% CI.

## Results

In total, 943 patients met the inclusion criteria with a mean follow-up period of 1 year. The mean age was 74.3 years and 50.4% were men. A total of 108 deaths (11.5%) were recorded within the follow-up period; eight (25%) for dabigatran compared with 44 (12.6%) for warfarin and 56 (9.9%) for aspirin. The mortality risk for patients on dabigatran was significantly higher than for patients on warfarin: HR = 2.1 (95% CI: 1.0 to 4.5), *P *= 0.05 after controlling for age. Aspirin had a lower (but not statistically significant) mortality risk compared with warfarin; HR = 0.75 (95% CI: 0.50 to 1.14), *P *= 0.18 after controlling for age.

## Conclusion

The results showed higher overall mortality in patients who presented to the ED with a bleeding complication and were taking dabigatran compared with warfarin or aspirin. Physicians should be aware of the potential higher mortality with dabigatran over warfarin when treating a bleeding patient. However, this was a single-centre retrospective analysis with a small number of patients taking dabigatran (*n = *32), and further studies are needed to corroborate the results.

